# Assessment of Internal Load and External Load in Senior Football Players: Differences Between Competitive Levels

**DOI:** 10.3390/jfmk11020242

**Published:** 2026-06-19

**Authors:** Diogo Tereso, José M. Gamonales, Víctor Hernández-Beltrán, Rui Paulo

**Affiliations:** 1 SPRINT—Sport Physical Activity and Health Research & Innovation Center|Centro de Investigação & Inovação em Desporto Atividade Física e Saúde|Portugal|FCT—Fundação Para a Ciência e a Tecnologia|Portuguese Foundation for Science and Technology, Polytechnic University of Castelo Branco, 6000 Castelo Branco, Portugal; diogotereso@hotmail.com (D.T.); ruipaulo@ipcb.pt (R.P.); 2 Faculty of Education and Psychology, University of Extremadura, 06006 Badajoz, Spain; 3 Training Optimization and Sports Performance Research Group (GOERD), Faculty of Sport Science, University of Extremadura, 10005 Cáceres, Spain; 4 Instituto Universitario de Investigación e Innovación en el Deporte (INIDE), Universidad de Extremadura, 10005 Cáceres, Spain

**Keywords:** football, performance, division, match demands, players

## Abstract

**Background:** Football is an intermittent sport characterized by high physical and physiological demands, which may be influenced by the competitive level. Understanding differences in match load is fundamental for optimizing training planning, fatigue management, and athlete performance and injury prevention. This study aimed to evaluate and compare external and internal load in senior football players in Portugal across five distinct competitive levels. **Methods**: Wimu Pro^TM^ (Hudl, Lincoln, NE, USA) and Garmin Heart Rate bands (Garmin International Inc., Olathe, KS, USA) were used to quantify and evaluate the external and internal load of the players. A total of 96 athletes were assessed, with ages ranging from 19 to 36 years (mean: 24.28 ± 4.72), who were divided into five competition levels (1st Division (n = 19), 2nd Division (n = 21), 3rd Division (n = 14), 4th Division (n = 20), and Regional Division (n = 22). **Results**: Significant differences were observed between competitive levels across several external load variables (*p* > 0.001). The 3rd Division and 4th Division showed higher values in variables associated with reactive and high-intensity actions (*p* < 0.001; effect size: 0.287), whereas the 2nd Division exhibited a more controlled load profile. Regarding internal load, significant differences were only observed in average heart rate during the second half (*p* = 0.043; effect size: 0.085), indicating distinct capacities to maintain physiological intensity under fatigue. **Conclusions**: It can be concluded that competitive level influences load profiles in football, although the differences do not follow a linear pattern. External and internal loads demonstrate greater discriminatory capacity between competitive levels than internal load.

## 1. Introduction

Football is characterized as an intermittent sport, combining high-intensity actions such as sprints, fast runs, and rapid changes in direction with periods of low intensity, such as walking or jogging, which require players to have a high level of physical, technical, tactical, and psychological preparation [1,2,3,4]. The variable and unpredictable nature of the game requires athletes to simultaneously possess well-developed aerobic and anaerobic capacities [5,6,7,8]. Understanding the physical demands imposed on players during matches is fundamental [4,9,10] as this information allows for more appropriate training planning, optimizes athlete recovery, contributes to a reduction in injury risk, and improves performance, both individually and collectively [8,11,12,13,14,15].

With technological advances, the monitoring of training and match load to which athletes are subjected has become increasingly common, both in competitive and training contexts [1]. This can be analyzed through two complementary dimensions [16]: external load (EL) and internal load (IL) [9,14]. EL refers to the physical stimuli imposed on the athlete (“work performed”) [17,18,19] and is generally quantified using technologies such as the global positioning system (GPS), which provide data on various parameters, such as distance covered, velocities, accelerations, and decelerations [20,21,22]. In the same line, some variables such as Player Load (PL) or impacts can be defined as neuromuscular variables, which are associated with the force executed by the athlete, as a result of the interaction with gravity and the teammates/opponents registered by triaxial accelerometers [23], and allow to identify the accumulated load of the player. On the other hand, IL refers to the athlete’s physiological and psychological response to these stimuli [14,19,20]. It is usually assessed using metrics such as heart rate (HR) [5,24] or the rating of perceived exertion (RPE) [25,26,27].

Integrated load monitoring, combining external and internal indicators, allows for a more comprehensive analysis of the physical demands of the game and provides an accurate and thorough characterization of the athlete’s load profile and condition [28]. Through this approach, it is possible not only to quantify the work performed but also to understand the actual impact that this effort has on the body [25,29,30,31]. Analyzing only one of these dimensions individually can lead to incomplete interpretations of the actual impact of training, especially because players with different physiological characteristics may respond differently to the same stimulus [19,32]. By integrating these two measures, coaches and sports science professionals can gain a more accurate understanding of training planning, optimize recovery strategies, and identify signs of excessive fatigue or risk of overtraining [19,30,33,34,35]. Previous studies suggest that players at higher competitive levels exhibit greater IL and EL values, both in training and matches, as well as physiological responses adapted to these demands [36,37], such as 20 m sprint (r = 0.61, *p* < 0.05) or dynamic stress load during official games (r = −0.57, *p* < 0.05).

Players at different competitive levels exhibit distinct physical, physiological, and tactical characteristics, which directly influence the load demands during training and competition [4,38]. Professional athletes tend to demonstrate greater aerobic and anaerobic capacity, higher maximal speeds, and increased tolerance to repeated efforts, resulting in more intense EL patterns. Consequently, their IL reflects more controlled and efficient physiological responses to the demanding stimuli encountered [39,40]. The literature has shown that elite players cover greater distances at high speed, perform more accelerations and decelerations, and sustain higher intensities during matches compared with players of lower competitive levels [9,41]. These differences can be explained by several factors, including the level of physical maturation [42,43], the experience accumulated in high-demand contexts [44,45], and higher quality and volume of training [46]. Thus, understanding how competitive level influences IL and EL is essential for correctly interpreting the demands of the game and adjusting training programs according to the specific profile of each group of players [47,48,49].

Despite the growing scientific interest in load monitoring in football, to date, there are no studies that simultaneously compare five distinct senior competitive levels using metrics normalized per minute, and integrated indicators of EL and IL were found. Understanding these differences may provide valuable information for coaches and strength and conditioning professionals, allowing researchers to tailor the training process to the specificities and constraints of each competitive level. Also, this information will help the training staff prevent injury through load management.

The present study aimed to assess and compare IL and EL indicators among senior football players in Portugal across five distinct competitive levels. Thus, it emerges as an original and necessary contribution, providing objective and relevant data on Portuguese football. This evaluation provides relevant information and allows the identification of response patterns and possible differences associated with performance level, contributing to a better understanding of the demands of football across different competitive levels.

## 2. Materials and Methods

### 2.1. Study Design

This study adopted an observational, cross-sectional, and comparative design, conducted in a real competitive context [50]. The investigation aimed to assess and compare IL and EL demands across different competitive levels in Portuguese football. Data were collected during official matches, with all variables measured at a single time point per match, without longitudinal follow-up [51].

As an observational study, no experimental manipulation was applied. The cross-sectional nature of the design allows for the comparison of match load profiles between competitive levels. This methodological approach is consistent with previous research in team sports performance and load monitoring [52,53].

### 2.2. Participants

Initially, 121 athletes agreed to participate in the present study. After the removal of missing values, dropouts, and incomplete cases, 96 athletes were included without excluding any field position. Athletes who played less than 60 min were excluded, as this was the protocol used to be eligible for inclusion in the present investigation.

A total of 96 athletes participated in the present study, belonging to five football teams from Portugal, with ages ranging from 19 to 36 years (mean: 24.28 ± 4.72). Table 1 shows the main characteristics of each level of competition.

### 2.3. Instruments

To carry out the present study, initially, a questionnaire of anamnesis (name, field position, injury history, and competitive level) was applied to define each of the groups and characterize the sample. It should be noted that the data were collected by the same team of researchers, adequately trained, using the defined protocols.

#### 2.3.1. External Load Measures

The EL was collected in official matches using an inertial device, WIMU PRO^TM^ (Hudl, Lincoln, NE, USA), which contains four triaxial accelerometers that detect and measure movement using a micro-electromechanical system with adjustable sample frequency of 10 to 1000 Hz, and the device was placed in a vest in the interscapular compartment (vertebral T2-T4 level). The data were stored in internal memory and later analyzed using SPRO software (v.990, RealTrack Systems, Almeria, Spain) [54,55,56].

#### 2.3.2. Internal Load Measures

The IL was assessed using HR during the match as the main variable. This variable was measured with a Garmin HR band (Garmin International Inc., Olathe, KS, USA), which sent data to the WIMU PRO device using Ant+ technology with a frequency of 4 Hz, and the device was attached to a chest strap positioned between the abdomen and the chest [54,55].

### 2.4. Variables

During the study, the following variables were recorded to quantify and evaluate the IL and EL of the player, considering each competition level. In addition, the load during training and matches was evaluated. Table 2 shows the main factor (dependent variables) selected and a definition of each one, as well as the unit used for its measurement. As well, the competition level was characterized as the independent variable. This variable was divided into five categories: 1st Division/2nd Division/3rd Division/4th Division and Regional Division.

### 2.5. Procedures

In the first phase, after selecting the teams that would participate in the study, the research objectives were presented to the group. All ethical procedures were considered, and the study was approved by the institution’s technical-scientific committee. Once the qualified players to participate in the tests were defined, written informed consent was obtained, and an anamnesis sheet was given to each of them. It should be noted that all ethical principles, norms, and international standards concerning the Helsinki Declaration and the Convention on Human Rights and Biomedicine will be followed, respected, and preserved [57]. It was approved by the Bioethics Committee of the University of Extremadura (Registration number 67/2017; 19 April 2017). Also, this investigation was developed under the Ethical Standards in Sport and Exercise Science Research [58]. The investigation respected the framework of Organic Law 3/2018 of 5 December on Personal Data Protection and guarantee of digital rights.

The assessments were carried out over two consecutive weeks at each of the clubs in the five different competitive levels. IL and EL load assessments were performed using WIMU PRO^TM^ (Hudl, Lincoln, NE, USA and Garmin HR bands (Garmin International Inc., Olathe, KS, USA), respectively. Two official matches for the respective championship were evaluated, one home game and one away game, and only athletes who played at least 60 min were included.

### 2.6. Statistical Analysis

#### 2.6.1. Preliminary Analysis

All collected data were grouped, and after assessing and identifying outlier values, these were excluded to minimize potential distortions in the results.

#### 2.6.2. Main Statistical Analysis

Descriptive statistics were performed for all analyzed variables, including mean and standard deviation. In the same line, all collected data were grouped, and after assessing and identifying outlier values, these were excluded to minimize potential distortions in the results. Then, a Shapiro–Wilk test was performed to analyze the data distribution, considering *p* > 0.05 as a normal distribution [59]. All data variables analyzed presented with a non-normal distribution. Kruskal–Wallis test was used to verify differences between groups. When significant differences were identified, multiple post hoc comparisons were conducted using Dunn’s test with Bonferroni correction. Finally, an effect size analysis was used to determine the magnitude of the effect, and effect size thresholds were interpreted as follows: 0.01 = small effect; 0.06 = moderate effect; 0.14 = large effect. The effect size was calculated using the epsilon squared value (ε^2^) [60]. All statistical analyses were performed using the SPSS software v. 29.0 (IBM, Chicago, IL, USA), and the significance level was set at *p* ≤ 0.05 to reject the null hypothesis [61].

## 3. Results

Table 3 presents the data regarding comparisons between the five groups in relation to the variables: Demographic and Anthropometric Characteristics, Kinematics EL, Neuromuscular EL, and Objective IL. In the category of Demographic and Anthropometric Characteristics, there are statistically significant differences regarding the variable Age when comparing 3rd Division players (20.09 ± 0.88) with all other competitive levels (*p* < 0.001; Effect Size = 0.146), with 2nd Division athletes showing the highest mean values (25.71 ± 4.37). Regarding the anthropometric variables Height and Body Mass, no statistically significant differences were observed, with 2nd Division athletes showing the highest mean values for both variables (182.90 ± 6.02 cm and 76.67 ± 6.34 kg, respectively).

In the category of Kinematics EL, the Distance (m/min) (*p* = 0.001; Effect Size = 0.119), the 4th Division level (112.17 ± 16.55) showed higher mean values than all other levels, while 2nd Division athletes had lower mean values compared with 1st, 3rd, and Regional Divisions players. Significant differences were found in Explosive Distance (m/min) (*p* < 0.022; Effect Size = 0.131) when comparing 4th Division athletes with those from the 1st, 2nd, and Regional Divisions, and when comparing 3rd Division athletes with those from the 2nd and Regional Divisions. The highest mean values were observed in 4th Division athletes (12.29 ± 2.66). For the variables Acc/min and Dec/min, the differences are similar (*p* < 0.001; Effect Size = 0.287 and *p* < 0.001; Effect Size = 0.285, respectively). Significant differences were observed when comparing 1st Division athletes with those from the 2nd, 4th, and Regional Divisions, and when comparing 3rd Division athletes with those from the 2nd, 4th, and Regional Divisions. The 4th Division athletes presented the highest mean values for both variables (38.22 ± 10.70 and 38.44 ± 10.85, respectively). Significant differences were observed for the variable HSR Abs (m/min) (*p* < 0.001; Effect Size = 0.287) when comparing 2nd Division athletes with those from the 1st, 3rd, and 4th Divisions, as well as when comparing Regional Division athletes with those from the 1st, 3rd, and 4th Divisions. Moreover, when comparing 3rd Division athletes with 4th and Regional Divisions athletes, the lowest mean values were observed. Finally, for the variable Avg. Speed (km/h), significant differences were observed (*p* = 0.005; Effect Size = 0.124) when comparing 3rd Division athletes with those from the 2nd and Regional Divisions, as well as when comparing 4th Division athletes with those from the 2nd and Regional Divisions. The highest mean values were observed in 3rd Division athletes (6.94 ± 0.53 km/h).

In the Neuromuscular EL category, statistically significant differences were observed among the groups for all variables: PL/min (*p* = 0.006; Effect Size = 0.090), PL/min 1st H (*p* = 0.03; Effect Size = 0.080), PL/min 2nd H (*p* = 0.001; Effect Size = 0.143), and Total Impacts/min (*p* = 0.024; Effect Size = 0.079). For the variable PL/min, 4th Division athletes (1.63 ± 0.26) exhibited higher mean values compared with athletes from the 2nd Division, 3rd Division, and Regional Division. For PL/min 1st H, statistically significant differences were observed when comparing 4th Division athletes with all other competitive levels, with 4th Division athletes presenting the highest mean values (1.68 ± 0.28). For the variable PL/min 2nd H, statistically significant differences were observed when comparing 2nd Division athletes with those from the 1st Division and 4th Division, and when comparing 4th Division athletes with those from the 3rd Division and Regional Division, with 4th Division athletes again presenting the highest mean values (1.72 ± 0.37). For the variable Total Impacts/min, 4th Division athletes (189.82 ± 26.04) exhibited the highest mean values and showed statistically significant differences compared with athletes from the 1st Division, 2nd Division, and 3rd Division.

Finally, in the Objective IL category, statistically significant differences were observed only for the variable Avg. HR 2nd H (*p* = 0.043; Effect Size = 0.085) when comparing 2nd Division athletes with those from the 1st, 3rd, and Regional Divisions.

**Figure 1:**
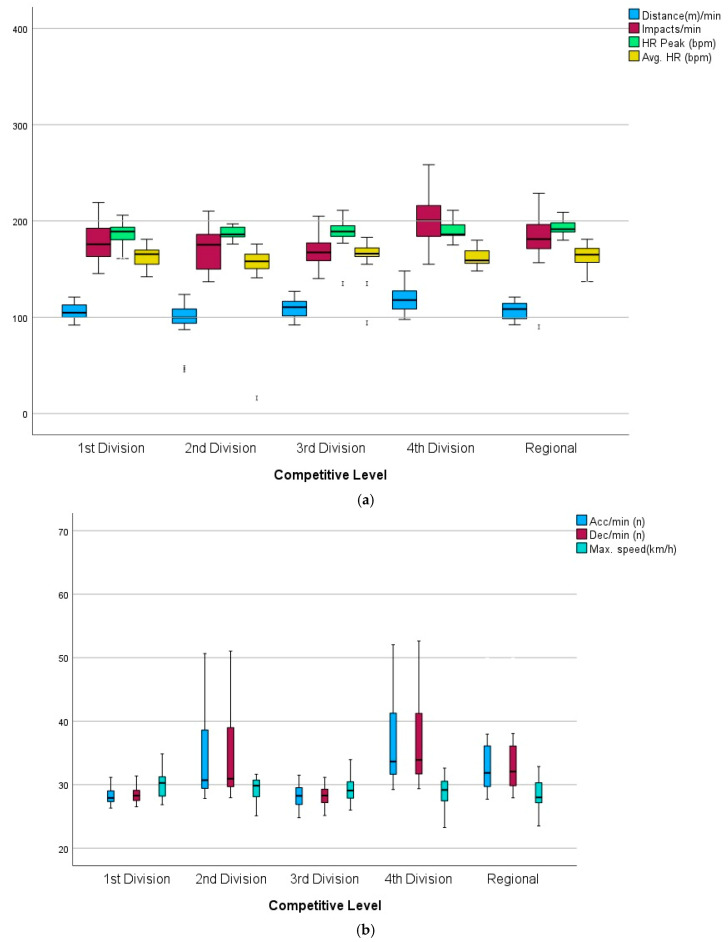
(**a**) Kinematics, neuromuscular, and internal variables boxplot. (**b**) Kinematics, neuromuscular, and internal variables boxplot.

Figure 1a,b, shows a box plot of the main variables analyzed, considering those that presented the most important results. Therefore, Figure 1a is related to the distance and impacts per minute, as well as some values such as HR peak and Avg. HR of the players. In the same line, Figure 1b, shows the main results of variables related to high-intensity actions, such as Max. speed, Accelerations, and Decelerations per minute.

## 4. Discussion

The present study aimed to evaluate the IL and EL of football players across the five competitive levels of Portuguese football. Generally, the different competitive levels exhibit distinct physical, physiological, and tactical profiles, which are directly related to the demands imposed on the athletes. In professional teams, such as those in the 1st Division, higher locomotor efficiency, enhanced ability to perform and sustain high-intensity actions, and a more structured style of play are typically observed, allowing for more effective game management. Although the 2nd Division is also professional, it tends to exhibit a more controlled style of play with less reliance on explosive actions. In contrast, intermediate or lower competitive levels, such as the 3rd, 4th, and Regional Divisions, typically display greater tactical variability and a higher number of transitions, giving rise to more reactive and less controlled game patterns. These differences directly influence the distribution of load to which athletes are exposed, reflecting the structural and functional characteristics of each competitive level. Considering these structural and physiological differences, the results of the present study provide a valuable opportunity to understand how load distribution is applied across different competitive levels. On the other hand, the existing literature reveals a scarcity of studies simultaneously comparing five competitive levels, which underscores the relevance and originality of the present study.

### 4.1. Demographic and Anthropometric Characteristics

Regarding the Age variable, the present study shows that there are statistically significant differences when the five competitive levels are compared, with 3rd Division athletes (20.09 ± 0.88) presenting the lowest mean age compared with all other levels. Studies in [62,63] corroborate the results found, also reporting the existence of significant differences between competitive levels. Conversely, studies conducted in other sports comparing male and female elite athletes did not find significant differences between different competitive levels [64,65]. In the present study, these differences may possibly be explained by the fact that the evaluated 3rd Division team is a reserve (B) team of a club competing in the 1st Division, resulting in a squad composed of a high number of young athletes who progress through the “B” teams before establishing themselves in the main teams.

For the variables Height and Body Mass, no statistically significant differences were observed when comparing any of the groups. Studies conducted by Tereso et al. [62] and by Chirosa-Rios et al. [64] in the sport of handball corroborate the results found. Conversely, studies conducted with futsal athletes [66], comparing soccer and basketball athletes [67] and comparing male and female elite athletes [65], found significant differences between distinct competitive levels. In the present study, athletes from the highest competitive levels showed the highest mean values for both variables, with 2nd Division athletes averaging 182.90 ± 6.02 cm in Height, and 1st Division athletes averaging 181.32 ± 6.72 cm. Similarly, for the Body Mass variable, the same trend was observed: the highest mean values were presented by athletes from the highest competitive levels, with 2nd Division athletes averaging 76.67 ± 6.34 kg, followed by 1st Division athletes with 75.64 ± 4.98 kg. Thus, although it is again the elite athletes (2nd and 1st Divisions) who present the highest mean values, which may reflect greater muscle development and better physical capacity, these two variables do not appear to be decisive factors for the competitive level [64,66,68].

### 4.2. Kinematics EL

For Distance (m/min), statistically significant differences were observed, with 4th Division athletes showing the highest mean values, whereas 2nd Division athletes presented the lowest mean values. This suggests that competitive level does not necessarily increase the intensity linearly (Regional Division > 2nd Division). In the 4th Division, the game pace may be higher and the style of play more direct, which could imply less tactical control compared with higher Divisions. Interestingly, the Regional Division values are very close to those of the 1st Division, which can possibly be interpreted as lower tactical organization, creating more space for running. However, some studies reported results contrary to the present article [25,39,69]. The study developed by Michaildis et al. [8], which evaluated athletes by field positions, did not find significant differences in the total distance covered throughout the game, but it did observe a decrease in all positions between the two halves. On the other hand, the study carried out by D’Elia [49] also found significant differences among the three competitive levels evaluated, reporting that professional athletes run on average 100–120 m/min. This is also observed in the study by Clemente et al. [70], which compared different playing positions, and in the present study, where all competitive levels showed values above 100 m/min, except for the 2nd Division. Although the growth pattern is not entirely linear in the present study, both studies indicate that different competitive levels exhibit distinct intensity levels.

For Explosive Distance (m/min), statistically significant differences were observed when comparing the groups. The study by Reynolds et al. [69] found different results from ours. This study compared three distinct competitive levels within the same team (U18, U23, and 1st Team) and did not find significant differences between the groups. Similarly, in that research, the first-team athletes showed relatively lower values compared with the other levels. The study by Santos et al. [71], which compared two distinct competitive levels (U-12 and U-15), did not find significant differences in the execution of SSGs (Small-Sided Games). In the present study, it is possible to conclude that the athletes with the highest mean values were those from 4th Division (12.29 ± 2.66), followed by 3rd Division athletes (12.00 ± 1.68). Conversely, the lowest mean values were observed in Regional Division athletes (10.04 ± 2.96). It was the intermediate and lower categories (3rd Division and 4th Division) that presented the highest values, possibly suggesting a more open style of play, with a greater number of transitions and reactive actions resulting in longer explosive movements. Conversely, the 1st and 2nd Divisions showed the lowest mean values, it could be reflected from better tactical organization, improved coordination between sectors, and more efficient management of high-intensity actions. Meanwhile, the Regional Division recorded the lowest mean values, possibly explained by physical limitations and a lower competitive pace.

For the variables Acc/min (n) and Dec/min (n), there is a direct relationship in the results, and the statistically significant differences are the same between group comparisons. That is, for Acc/min (n), the highest mean values were observed in 4th Division athletes (38.22 ± 10.70), while the lowest mean values were observed in 3rd Division athletes (27.71 ± 1.47). For Dec/min (n), the pattern was the same, with 4th Division athletes showing the highest mean values (38.44 ± 10.85) and 3rd Division athletes the lowest (27.90 ± 1.39). In a previous study, no significant differences were found between the evaluated levels, although the mean values presented are very close to those found in the present study [25]. The values observed for 4th Division may suggest a more reactive, chaotic, and direct style of play, with a greater number of speed changes. In contrast, the 1st Division presented one of the lowest mean values. On the other hand, the Regional Division level presented intermediate values, possibly explained by a less structured competitive pace. Thus, it is possible to conclude that acceleration and deceleration actions seem to depend more on the tactical characteristics and dynamics of the style of play than on the competitive level of each team.

For the variable HSR Abs (m/min), statistically significant differences were observed between competitive levels, but no linear pattern was revealed. That is, it would be expected that higher levels would possibly present greater HSR values. The study by Branquinho et al. [25] compared U18 and U19 athletes, and although no significant differences were found between the groups, the older athletes presented higher mean values. On the other hand, the study by Clemente et al. [69] which compared three distinct competitive levels within the same team (U18, U23, and 1st Team), found results similar to ours, with significant differences among the three levels and the highest mean values observed in the intermediate competitive level. This pattern was also observed in the present study, where the highest mean values were recorded for 3rd Division athletes (9.26 ± 2.76), followed by 1st Division athletes (8.07 ± 2.83). Although the 1st Division values are close to the highest, reflecting the high physical capacity and execution speed characteristic of elite levels, the Regional Division level presented the lowest mean values (4.64 ± 2.59), reflecting lower competitive intensity. It is the 3rd Division athletes who show the most striking results, possibly suggesting a style of play with a higher number of transitions and high-intensity actions, potentially due to lower tactical organization and greater available space. Thus, we understand that high-intensity distance covered does not depend solely on the competitive level. The study by Datson et al. [72] reports that male football players perform on average 30% more high-intensity movements compared with female football players.

The results for Max. Speed (km/h), although not showing statistically significant differences when comparing the groups, demonstrate a clear trend of differentiation between competitive levels. D’Elia [49] presents similar results, where athletes from the higher competitive level showed the highest mean values, although no significant differences were observed among the three evaluated levels. Conversely Pérez-Contreras et al. [39] which compared adult athletes with U-19 athletes, found significant differences, with U-19 athletes presenting the highest mean values. On the other hand, García-Ceberino et al. [73] found significant differences among the evaluated groups (Total, Training Match, SSGs, and Analytical Task), with the highest mean value observed in the Training Match. In the present study, 1st Division players presented the highest mean values (30.53 ± 2.18), followed by a gradual decrease across the other levels, highlighting greater sprinting capacity, typical of professional contexts. This trend is consistent with the existing literature, which reports that elite athletes perform high-intensity actions more frequently and reach higher speeds compared with players from lower levels. Sprinting can thus possibly be understood as a distinguishing capacity of elite athletes.

Avg. Speed (km/h) showed statistically significant differences between competitive levels, with the highest mean values observed in 3rd Division athletes (6.94 ± 0.53), possibly reflecting a generally faster overall pace. The 1st Division and 4th Division also presented relatively high values, consistent with the physical demands of these competitive contexts. On the other hand, the 2nd Division showed relatively low mean values (6.25 ± 0.94), possibly associated with a more compact and tactical style of play, with fewer prolonged runs. The lowest competitive level (Regional Division) presented a moderate average speed (6.34 ± 0.78), possibly reflecting lower technical quality and greater variability in game pace. Again, these results reinforce that average speed does not relate linearly to competitive level. Branquinho et al. [25] assessed Avg. Speed differently (m·min^−1^) and found results different from those of the present study. García-Ceberino et al. [73] also found significant differences among the evaluated groups (Total, Training Match, SSGs, and Analytical Task), with the highest mean value observed in the Training Match.

### 4.3. Neuromuscular EL

For the variable PL/min, statistically significant differences were observed among the different competitive levels [49]. The study by Gamble et al. [74] which assessed Gaelic Football athletes It reported a considerably higher PL (9.0 ± 1.9) and found significant differences between the different field positions. Similarly, García-Ceberino et al. [73] found significant differences among the evaluated groups (Total, Training Match, SSGs, and Analytical Task), with the lowest mean value observed in the SSGs. The highest mean values were observed in 4th Division athletes (1.63 ± 0.26). These results suggest a particularly demanding game context from a neuromuscular perspective, which may reflect a very physical style of play. This aligns with observations made in other variables (accelerations, decelerations, and average speed), reinforcing the idea of a physically more demanding and possibly less structured competition. The 3rd Division (1.48 ± 0.22) and 1st Division (1.47 ± 0.34) showed very similar mean values, reflecting high-intensity patterns, but possibly more organized and associated with greater technical-tactical quality. The Regional Division presented an intermediate value (1.41 ± 0.36), consistent with a less structured and physically more variable style of play. Finally, the 2nd Division presented the lowest mean values (1.35 ± 0.33), suggesting a more controlled style of play with fewer demanding neuromuscular actions. This aligns with the pattern observed so far, where this Division consistently shows the lowest values in intensity-related variables, possibly indicating a more tactical style of play, with fewer explosive actions and greater control.

For the variable PL/min 1st H, statistically significant differences were observed among the evaluated groups. The 4th Division once again recorded the highest mean values (1.68 ± 0.28), indicating a game start with a higher neuromuscular load and an intense pace of short, explosive actions. The 3rd Division (1.49 ± 0.25) and 1st Division (1.48 ± 0.34) again showed very similar mean values, reflecting high and consistent intensity patterns. The Regional Division (1.47 ± 0.38) appeared just behind the 3rd and 1st Divisions, reflecting a less structured but equally demanding neuromuscular style of play. The 2nd Division again presented the lowest values (1.40 ± 0.37), maintaining the trend of being the category with the lowest neuromuscular intensity. Gamble et al. [74], reported a considerably higher PL (9.1 ± 2.1) and found significant differences between different field positions, although no differences were observed between the first and second halves.

For the variable PL/min 2nd H, statistically significant differences were observed among the five evaluated groups. The highest mean values continued to be observed in 4th Division (1.72 ± 0.37), indicating a second half that is even more demanding from a neuromuscular perspective, possibly associated with greater alternation in game pace, higher levels of fatigue, and potentially a greater number of physical duels in the final phase of the game. This increase from the first to the second half may suggest a reduced capacity for effort management. Barret et al. [75] observed an increase in PL overtime was also observed, which was likely attributed to changes in strategy and/or reduced locomotor efficiency. In the same line, Gamble et al. [76] found significant differences between the two halves. In the 1st Division, an increase from the first to the second half was also observed (1.55 ± 0.37), which may reflect the athletes’ physical capacity to not only maintain but even increase neuromuscular load in the final moments of the game. The 3rd Division (1.49 ± 0.27) presented intermediate values, consistent with high-intensity patterns. The Regional Division presented lower mean values in the second half compared with the first half (1.39 ± 0.35), suggesting a greater physical drop-off and lower consistency in intensity throughout the game. The 2nd Division again showed the lowest mean values (1.34 ± 0.31), reinforcing the profile of a more controlled style of play with fewer high-intensity actions.

It is important to emphasize that differences in PL/min between the first and second halves should not be interpreted using the same criteria across different competitive levels. In the 1st Division, this increase may reflect a strategic intensification of game pace, greater capacity for effort management, and the influence of substitutions, which tend to raise intensity in decisive moments. In the 2nd Division, a decrease was observed, possibly explained by a more controlled style of play and a lower reliance on explosive actions, regardless of the game phase. In the 3rd Division, no fluctuations were observed; this consistency may reflect a balance between physical demand and the ability to manage game pace. In 4th Division, the observed increase could be explained by lower game control, leading to more reactive actions, that is, higher intensity but less controlled. In the Regional Division, the decrease may possibly reflect accumulated fatigue and, consequently, a reduced capacity to repeat high-intensity actions.

For the variable Total Impacts/min, statistically significant differences were observed. Opposite results were found in the study developed by D’Elia [49]. The 4th Division athletes (189.82 ± 26.04) presented the highest mean values according to the number of impacts; these values may reflect a more chaotic game context or a high number of impacts during game actions. The 3rd Division (172.60 ± 19.83) and 1st Division (172.27 ± 27.29) showed moderate values, reflecting high but controlled intensity. The 2nd Division (166.22 ± 32.57) presented the lowest mean values, possibly characteristic of a more structured style of play, which, although physically demanding, also exhibits less motor variability. The Regional Division (176.62 ± 31.62) showed the second highest impact values, reflecting lower tactical organization and reduced capacity to manage effort. These differences may possibly explain that competitive level influences the quantity and quality of impact actions during the game.

### 4.4. Objective IL

Max. HR did not show statistically significant differences between competitive levels, with mean values ranging from 187.60 to 193.00 bpm. This homogeneity suggests that maximal cardiovascular capacity is similar, regardless of competitive level. Max HR is typically determined by individual factors and is less influenced by game dynamics [39]. This is supported by the present study developed by Gamble et al. [74] reporting mean values very similar to those of the present study, but also without significant differences between the assessed groups (field positions). Michaildis et al. [8] evaluated % HRmax across different field positions and found no significant differences. In the present study, 3rd Division athletes (193.00 ± 9.98) showed the highest mean values, which may possibly be explained by the individual factor of being younger athletes. The Regional Division presented a relatively high mean value (190.82 ± 10.53), these differences appear to reflect variations in game patterns rather than physiological differences between groups. The professional competitive levels, 1st Division (188.37 ± 9.71) and 2nd Division (187.95 ± 6.31), showed similar mean values, suggesting a lower need to reach maximal thresholds to meet game demands. Thus, the results reinforce that Max. HR is not a discriminatory factor between competitive levels.

Avg. HR did not show statistically significant differences between competitive levels, with mean values ranging from 151.43 to 167.61 bpm. This similarity between groups suggests that the overall physiological intensity of the game is relatively consistent across different competitive levels and cannot be considered a discriminatory factor of competitive level. Pérez-Contreras et al. [39] support the present study, which also did not find significant differences between the evaluated groups. The 3rd Division (167.61 ± 9.69) presented the highest mean values, possibly explained by the younger age of the athletes, but also characteristic of a more direct style of play with a higher number of transitions. This is also consistent with other findings, including higher HSR, greater Avg. Speed, and many impacts. In contrast, the 2nd Division (151.43 ± 32.45) presented the lowest mean values. This result reinforces the pattern observed across almost all variables, characteristic of a more controlled style of play with fewer high-intensity transitions. The 1st Division and the Regional Division showed similar values (162.50 ± 9.68 and 163.00 ± 12.69, respectively), although likely for different reasons: in the 1st Division, greater technical-tactical efficiency, and in the Regional Division, higher overall effort. The 4th Division (159.70 ± 8.02) presented intermediate mean values, characterizing its competitive dynamics with moderate-to-high intensity. In summary, Avg. HR reflects overall intensity rather than the specific actions associated with competitive level. Avg. HR 1st H did not show statistically significant differences between groups, with mean values ranging from 160.55 to 166.47 bpm. These results indicate that the initial physiological intensity of the game is similar across groups, suggesting that the average cardiovascular response in the first half does not effectively determine competitive level. The 3rd Division (166.47 ± 21.38) presented the highest mean values, which may reflect a more intense start to the game, while the 1st Division (160.55 ± 13.67) showed the lowest mean values, possibly explained by greater tactical efficiency and the ability to manage the initial game pace. The 2nd Division (163.45 ± 9.82), 4th Division (165.00 ± 12.07), and Regional Division (164.86 ± 10.75) showed similar mean values, indicating comparable physiological patterns in the first half. Once again, it can be concluded that Avg. HR 1st H is influenced primarily by game dynamics rather than competitive level. Gamble et al. [74] reported mean values (165 ± 11) like those of the present study and found differences between the first and second halves. These results are consistent with the literature, which shows that in the first half, Avg. HR reflects a standardized overall game pace.

The variable Avg. HR 2nd H was the only one to show statistically significant differences in the Objective IL category. Mean values ranged from 153.43 to 163.45 bpm. The 3rd Division (163.45 ± 11.26) and 1st Division (163.36 ± 8.96) presented the highest mean values, suggesting greater physiological intensity in the second half. In contrast, the 2nd Division (153.43 ± 12.93) showed the lowest mean values, reinforcing the trend previously observed in other variables and possibly indicating a more controlled style of play with fewer continuous high-intensity efforts. The 4th Division (158.72 ± 12.35) and the Regional Division (160.46 ± 15.26) showed intermediate values, reflecting lower fatigue management efficiency and greater tactical variability. These results suggest that physiological intensity in the second half may possibly discriminate more clearly between different competitive levels, reflecting each team’s capacity to maintain or increase intensity under more demanding situations. It can be considered that the second half, compared with the first half, better discriminates competitive levels. The fact that statistically significant differences were found only in the second half indicates that the ability to manage fatigue, intensity, and responses to the final demands of the game differs between levels. Furthermore, Gamble et al. [74] reported mean values (160 ± 10) like those of the present study and also found significant differences between the first and second halves.

### 4.5. Limitations

The main limitation is that the study used a transversal design, which prevents analysis of the evolution of the workload over the whole season. This prevents the establishment of relationships between other contextual variables such as the player position, the number of scores and the location of the match (local or visitant), because these variables can directly influence the sports performance. Also, the analyses focused only on objective variables related to the IL and EL, without analyzing subjective variables such as the RPE or wellness. In the same line, different variables such as playing position, match location, opponent strength, score-line, tactical system, match status, and substitutions were not evaluated and considered as variables that can influence the sports performance.

On the other hand, another limitation was the small number of players from the 3rd Division. This can influence the results in order to compare with the other categories or levels. However, in order to prevent or reduce the risk of bias, all the variables were normalized to the minute to facilitate the comparison between categories and extract relevant conclusions.

### 4.6. Strength and Future Research Lines

This study is one of the first to analyze the IL and EL of the players and compare them across five competition levels in Portugal. Also, the inertial devices used allowed to quantify the EL of the players in a very accurate way. The normalization of the variables allowed for a proper comparison of the competition levels and identified great differences in sports performance. As future research lines, it is recommended to develop a longitudinal design in order to analyze the workload over a season or various seasons. This will help the coaching staff to analyze the training and match load and adapt the tasks considering the player’s threshold. In the same line, it could be beneficial to add a position analysis and the IL and EL of each position to prevent injury incidence.

### 4.7. Practical Application

From an applied perspective, the findings enable coaches to adapt training planning and load management strategies according to competitive level, highlighting the greater discriminatory capacity of external and neuromuscular load compared with internal load, and emphasizing the importance of the second half as an indicator of the ability to manage fatigue in demanding competitive contexts.

The HSR is a key factor that reflect the number of actions with an upper speed than 21 km/h, which is a result of a high-intensity actions that can derived in high fatigue and incomplete recovery. Therefore, the coaching staff must develop task with low number of sprints in sessions pre- and post-match, exactly in sessions MD-1 and MD+1 to facilitate the recovery after the efforts. Considering the PL of the players, the high results were derived from a great number of transitions during the game, change or directions and pace and a high load accumulative during the match. Therefore, the coach must consider applying strategies focused on a tactic game to reduce the number of high actions, better management of the pace and to reduce the number of unnecessary explosive actions.

As a summary, the coaches must understand that high load in the tasks it is not related to obtain a better performance on the player, it can be derived in injuries or overloading. Therefore, they have to control the exertion and the efficient due to this factor are key as a performance indicator. Finally, the second half is a key factor to identify the differences of each category.

## 5. Conclusions

The results show that competitive level significantly influences the workload profile to which players are exposed, although these differences do not follow a linear pattern across competitive levels.

In general, EL variables showed notable differences between competitive levels. These differences can be derived from distinct playing styles, levels of tactical organization, and competitive demands. While the 1st Division demonstrated better aerobic fitness and the ability to manage intensity throughout the game, intermediate levels such as the 3rd and 4th Divisions showed higher metrics associated with high-intensity actions, such as accelerations and decelerations. The 1st Division, in turn, stood out for a more controlled style of play with lower demands across most variables, whereas the Regional Division showed greater difficulty in managing and resisting fatigue, especially in the second half, due to the high number of high-intensity actions during the match, resulting in a decrease in the PL and the HR values from the first to the second half.

Regarding IL, it can be considered that it is not a discriminatory factor between competitive levels. However, mean HR in the second half showed significant differences, suggesting that the ability to maintain intensity under fatigue may constitute a differentiating factor between competitive levels.

Thus, these results reinforce the importance of an integrated approach to load monitoring for a more comprehensive understanding of the demands of the game. Also, this load management must be considered by all the coaching staff to reduce the probability of injury and manage properly the competitive performance of the player during the whole season and reduce the risk of being overloaded.

Despite the limitations, this research contributes to future studies exploring the evolution of load throughout the season, the influence of playing position, and the relationship between load, performance, and injury risk.

## Figures and Tables

**Table 1:** Characteristics of the sample.

Competition Level	n	Age (Mean and SD) (Years)	Training Sessions
1st Division	19	24.32 ± 4.20	5 to 6 training sessions per week of 90 min
2nd Division	21	25.71 ± 4.37	5 to 6 training sessions per week of 90 min
3rd Division	14	20.00 ± 0.88	5 to 6 training sessions per week of 90 min
4th Division	20	25.10 ± 5.24	4 to 5 training sessions per week of 90 min
Regional Division	22	24.86 ± 5.17	3 to 4 training sessions per week of 90 min

**Table 2:** Main characteristics of the variables used.

Type of Variable	Instrument	Variable	Definition	Unit of Measurement
Demographic and Anthropometric Characteristics	Questionnaire	Age	Number of years of each player	year
		Height	The height of each player	cm
		Body Mass	The weight of each player	kg
Kinematics EL	Inertial device	Distance	Total distance covered	m
		Explosive Distance	Distance covered with an acceleration greater than 1.12 m/s^2^	m
		Acc/min	Number of accelerations achieved	n
		Dec/min	Number of decelerations achieved	n
		HSR	Number of meters the player has exceeded 17.6 km/h	m
		Max. speed	Maximum speed achieved	(km/h)
		Avg. speed	Average of speed during the whole match	(km/h)
Neuromuscular EL	Inertial device	PL/min	Vector sum of accelerations recorded by the device in its three axes	au
		PL/min 1st H	Vector sum of accelerations recorded by the device in its three axes in the first half	au
		PL/min 2nd H	Vector sum of accelerations recorded by the device in its three axes in the second half	au
		Total impacts/min	Vector sum of the forces G supported by a player in all three planes	G
Objective IL	HR bands	Max. HR	Maximum value reached in beats per minute during the session	bpm
		Avg. HR	Average beats per minute	bpm
		Avg. HR 1st H	Average beats per minute in the first half	bpm
		Avg. HR 2nd H	Average beats per minute in the second half	bpm

Note. cm: centimeters; kg: kilograms; m: meters; n: numbers; km/h: kilometers per hour; au: arbitrary unit; G: G force; bpm: breath per minute; PL: player load; HSR: high speed running; H: half; HR: heart rate; EL: external load; IL: internal load; Acc: accelerations; Dec: decelerations; Max.: maximum; Avg.: average.

**Table 3:** Comparisons between groups (competitive level) regarding the variables.

Variables		1st Div (n = 19)		2nd Div (n = 21)		3rd Div (n = 14)		4th Div (n = 20)		Regional Division (n = 22)		*p*	ε^2^	Post Hoc
		Mean	SD	Mean	SD	Mean	SD	Mean	SD	Mean	SD			
Demographic and Anthropometric Characteristics	Age	24.32	7.39	25.71	4.37	20.09	0.88	25.10	5.24	24.86	5.17	<0.001 *	0.146	3rd Div < All
	Height (cm)	181.32	6.72	182.90	6.02	180.43	5.63	180.00	8.03	180.50	5.86	0.439	0.025	
	Body Mass (Kg)	75.64	4.98	76.67	6.34	73.64	4.77	73.00	9.83	74.11	4.46	0.164	0.044	
Kinematics EL	Distance (m)/min	104.70	7.39	96.10	18.13	106.06	8.05	112.17	16.55	103.57	17.72	0.001 *	0.119	2nd Div < 1st Div, 3rd Div and Region; 4th Div > 1st Div, 2nd Div, 3rd Div and Region
	Explosive dist. (m)/min	10.91	1.78	10.27	2.60	12.00	1.68	12.29	2.66	10.04	2.96	0.022 *	0.131	3rd Div > 2nd Div and Region; 4th Div > 1st Div, 2nd Div and Region
	Acc/min (n)	28.40	1.38	34.54	6.96	27.71	1.47	38.22	10.70	33.21	5.68	<0.001 *	0.287	1st Div < 2nd Div, 4th Div and Region 3rd Div < 2nd Div, 4th Div and Region
	Dec/min (n)	28.56	1.38	34.76	6.96	27.90	1.39	38.44	10.85	33.42	5.71	<0.001 *	0.285	1st Div < 2nd Div, 4th Div and Region 3rd Div < 2nd Div, 4th Div and Region
	HSR Abs. (m)/min	8.07	2.83	5.23	2.49	9.26	2.76	7.19	3.18	4.64	2.59	<0.001 *	0.287	2nd Div < 1st Div, 3rd Div and 4th Div; 3rd Div > 4th Div; Region < 1st Div, 3rd Div and 4th Div
	Max. speed(km/h)	30.53	2.18	29.19	2.24	29.43	2.19	28.85	2.42	28.53	2.30	0.087	0.084	
	Avg. speed(km/h)	6.68	0.41	6.25	0.94	6.94	0.53	6.80	0.82	6.34	0.78	0.005 *	0.124	3rd Div > 2nd Div and Region; 4th Div > 2nd Div and Region
Neuromuscular EL	PL/min	1.47	0.34	1.35	0.33	1.48	0.22	1.63	0.26	1.41	0.36	0.006 *	0.090	4th Div > 2nd Div, 3rd Div and Region
	PL/min 1st H	1.48	0.34	1.40	0.37	1.49	0.25	1.68	0.28	1.47	0.38	0.03 *	0.080	4th Div > all
	PL/min 2nd H	1.55	0.37	1.34	0.31	1.49	0.27	1.72	0.37	1.39	0.35	0.001 *	0.143	2nd Div < 1st Div and 4th Div; 4th Div > 3rd Div and Region
	Total impacts/min	172.27	27.29	166.22	32.57	172.60	19.83	189.82	26.04	176.62	31.62	0.024 *	0.079	4th Div > 1st Div, 2nd Div and 1st Div
Objective IL	Max. HR (bpm)	188.37	9.71	187.95	6.31	193.00	9.98	187.60	6.72	190.82	10.53	0.586	0.055	
	Avg. HR (bpm)	162.50	9.68	151.43	32.45	167.61	9.69	159.70	8.02	163.00	12.69	0.091	0.093	
	Avg. HR 1st H (bpm)	160.55	13.67	163.45	9.82	166.47	21.38	165.00	12.07	164.86	10.75	0.525	0.020	
	Avg. HR 2nd H (bpm)	163.36	8.96	153.43	12.93	163.45	11.26	158.72	12.35	160.46	15.26	0.043 *	0.085	2nd Div < 1st Div, 1st Div and Region

SD: standard deviation; EL: external load; IL: internal load; Acc: accelerations; Dec: decelerations; HSR: high speed running; PL: player Load; HR: heart rate; SD: standard deviation; * *p* < 0.05; ε^2^: epsilon squared.

## Data Availability

The original contributions presented in this study are included in the article. Further inquiries can be directed to the corresponding authors.
